# Association between Family Structure and Physical Activity of Chinese Adolescents

**DOI:** 10.1155/2016/4278682

**Published:** 2016-03-30

**Authors:** Lijuan Wang, Jing Qi

**Affiliations:** ^1^Shanghai University of Sport, Shanghai 200438, China; ^2^Beifang University of Nationalities, YinChuan 750021, China

## Abstract

*Background*. This study examines the association between family structure and moderate-to-vigorous physical activity (MVPA) of adolescents in China.* Methods*. The participants included 612 adolescents (317 boys and 295 girls) from Shanghai with ages ranging from 10 to 16 years. Accelerometers were used to measure the duration of MVPA of adolescents, and questionnaires on family structure were completed by the parents of these adolescents.* Results*. Findings suggested that family structure significantly increased the likelihood of adolescents engaging in physical activity (PA) and explained 6% of MPVA variance. Adolescents living in single-parent households and step families were more physically active than those living in two-parent homes and with biological parents, respectively. However, adolescents residing with grandparents were less active than those living with neither grandparent. No significant difference was found in MVPA time between adolescents living with one sibling and those without siblings.* Conclusion*. Family environment may be considered in the development of PA interventions and policies, and adolescents living with their grandparents may be targeted in PA promotion.

## 1. Introduction

Regular physical activity (PA) participation is an important contributor to adolescent health [1]. Evidence linking PA and health specifically suggests that regular PA has beneficial effects on the adiposity, blood lipids and lipoproteins, bone health, and psychological well-being of adolescents [1]. The World Health Organization (WHO) developed a set of PA guidelines to encourage participation and recommended that children and adolescents engage in at least 60 minutes of daily moderate-to-vigorous physical activity (MVPA) [2]. However, evidence suggests that Chinese adolescents do not adhere to the recommended guideline [3–5]. The China Health and Nutrition Survey, which was conducted among nearly 2700 youth aged 6 to 18 years from eight provinces in China, shows that 72% of Chinese youth engaged in in-school moderate-to-vigorous PA (MVPA) for 90 to 110 min/week; however, only 8% were engaged in MVPA outside of school [4]. Wang et al. measured the MVPA of 2163 students in the fourth to 11th grades from 11 cities in China and found that Chinese city children and adolescents spent an average of 28 min/day engaged in MVPA and 521 min/day engaged in sedentary behavior [5]. Factors associated with these behaviors need to be well understood to develop effective strategies to promote PA among Chinese children and adolescents as well as to provide a guide for targeted interventions [6, 7].

Family is an important socializing agent that has a positive influence on the initiation and maintenance of adolescents' health-related PA habits [8]. However, only Bagley et al. explored how five family structure variables influenced PA and television viewing time of children and found a significant relationship between one variable (i.e., having siblings or not) and PA in girls. Other variables of family structure were not associated with PA of children [9]. Limited number of studies and inconsistent findings of the study implied that more studies are necessary to determine the relationship between family structure and the PA level of children and adolescents. On the other hand, existing studies that investigated the relationship between family structure and the PA level of children and adolescents considered the family structure based on having siblings or not but did not include a broad definition of the household structure (e.g., step families and lineal joint families). Family structure measurement is complex, and a limited view of the structure may impede understanding of its influence on children's behaviors. Gustafson and Rhodes reviewed studies on the correlates of the PAs of children and adolescents and suggested that studies with more specific family structure classifications are necessary [10].

Traditional Chinese families have been facing both internal and external challenges since the late 1970s. China implemented the reform and opening up policy and the one-child policy during this period. A few significant changes in the family structure can be noted with the socioeconomic development and change of people's value [11, 12]. First, the number of single-parent and step families is increasing because people have become more tolerant of divorce and remarriage. Second, most families have only one child because of the implementation of the one-child policy over the past two decades [11, 12]. Third, many families have grandparents that care for grandchildren in both urban and rural China; this phenomenon has resulted in the popularity of the lineal joint families [13]. The fifth Census in China (2010) shows that 65.3% of the families in China are nuclear (i.e., with two parents and children), 15.3% are lineal joint, 17.0% are one-person, 0.7% are single-parent, 0.4% are remarried, and 1.3% are other families. However, no study has explored the association between the various types of family structure and the PA levels of children and adolescents in China. Hence, the current study aims to bridge this research gap by determining the associations among family structure and PA of Chinese children aged 10–16 years while accounting for background demographic factors such as age, gender, and socioeconomic status (SES).

## 2. Method

### 2.1. Participants

Three primary schools and four secondary schools were selected randomly from four districts of Shanghai, a city located in eastern China. The principals of the seven schools were contacted, and their consent to be included in the study was obtained. All students in Grades 5 to 6 of the primary school and all students of Grades 7 to 8 in secondary schools were invited to participate in this study. A total of 964 out of 1362 students agreed to participate. The university ethics committee and relevant educational authorities approved the current study.

Among the total 964 participants, 352 were excluded based on the following exclusion criteria: (1) About 26 students were excluded because of accelerometer malfunction or loss. (2) About 254 students were excluded because their valid accelerometer data did not cover at least two valid weekdays and one valid weekend day. (3) About 72 participants were subsequently eliminated because of incomplete survey. The final analysis sample consisted of 612 participants with a response rate of 64.8%.

### 2.2. Measures

#### 2.2.1. Adolescents' MVPA Level

MVPA of adolescents in the present study was measured using Actigraph GT3X Activity Monitors. The accelerometer has been shown to be a valid, reliable, and objective tool for assessing MVPA of children and adolescents [14]. The children were asked to wear the monitor for seven consecutive days during their waking hours, removing them upon bathing, taking a shower, or swimming [15]. All accelerometers were charged and programed to record PA in 30-second epochs. After the test, the original Actigraph data files were downloaded to a personal computer using ActiLife software 6.5.2. A recording of more than 20,000 counts per minute (CPM) was considered potential accelerometer malfunction and the corresponding data excluded from the analysis [5]. Accelerometer data were considered valid if more than 600 minutes (10 hours) of monitoring per day (excluding strings of zeros for 20 minutes or longer) was recorded over the entire monitoring period [16]. The accelerometer data were included in the final analysis if they contained at least two valid weekdays and one valid weekend day [5]. The Chinese specific cutoff points were used to determine the activity level thresholds [17]. These points were more suitable for Chinese children compared with previously developed cutoff points, which defined MPA as 2,800 CPM to 3,999 CPM and VPA as 4,000 CPM [17]. Finally, the MVPA levels were reported as daily time (minutes/day) spent in MVPA across all valid days.

#### 2.2.2. Family Structure

Family structure was assessed using an open-ended question that asked parents to list all individuals who have lived with their children at least part of the time and have been considered family. They were also asked about these individuals' relationship with their children (e.g., father and mother). Based on their frequency and conceptual importance, open-ended responses were coded by the researcher and a research assistant into various subcategories according to four family structure variables such as number of parents, nature of parents, number of grandparents, and number of siblings. Number of parents was collapsed into three categories: no parents, single-parent, and both parents. Nature of parents was categorized as living with biological parents, step parents, and adopted parents or relatives. The other two family structure variables were the number of grandparents which was dichotomized as no grandparents and being with one or two grandparents and the number of siblings which was analyzed into two ways: no siblings and one sibling.

#### 2.2.3. Other Control Measures

The models in the present study include the control variables of gender (boys = 0, girls = 1), age which is measured in years, and SES. The Hollingshead Four-Factor Index was used to determine SES in the present study [18, 19]. Using the index, SES was calculated based on education, occupation levels, sex, and marital status. Parental education categories were coded from low to high (1–7): 1, lower than Grade 7; 2, junior high school level (Grade 9); 3, partial high school (Grade 10 or 11); 4, high school graduate; 5, partial college or specialized training; 6, standard college or university graduation; and 7, graduate professional training (graduate degree). Occupational status was assessed by asking the participants about the type of work that they spent the most time on in their everyday life. We coded these open-ended responses based on the following census categories: 1, farm laborers/menial service workers; 2, unskilled workers; 3, machine operators and semiskilled workers; 4, small business owners, skilled manual workers, craftsmen, and tenant farmers; 5, clerical and sales workers, as well as small farm and business owners; 6, technicians, semiprofessionals, and small business owners; 7, small business owners, farm owners, managers, and minor professionals; 8, administrators, minor professionals, and proprietors of medium-sized businesses; and 9, high executives, proprietors of large businesses, and major professionals. The SES score of an individual was calculated by multiplying the scale value for occupation by weight of five and the scale value for education by a weight of three. This scale considers the differences in the ways adult family members participate in the economic system. For example, when both parents are gainfully employed, the education and occupation scores for the mother and father were added and divided by two. Specific guidelines are provided for handling data for separated, divorced, or widowed individuals [20]. Finally, SES index values (range: 8–66) were categorized as high (values of 48–66), middle (values of 28–47), or low (values of 8–27) [20].

### 2.3. Data Collection Procedure

Data were collected by the researcher and two research assistants. The researchers introduced the objectives and methods of the study to the participants before data collection. The research assistants obtained informed consent from all participating adolescents and their parents. The adolescents were provided with accelerometers and instructed to wear the instrument under their clothes by fastening it to their right hipbone using an elastic belt for seven consecutive days. The accelerometers were initialized using ActiLife software 6.5.2 to begin collecting data at 0:00. The participants were instructed to continue with their normal daily routines during the monitoring period. Written instructions were also given to all participants and their parents to remind them to wear the accelerometer to increase compliance. The adolescents were required to return the accelerometers after eight days to ensure the seven days of complete data collection. Thereafter, SES and family structure survey was administered to the participating adolescents with valid accelerometer data. The questionnaire was brought to their parents for completion to ensure accuracy of the survey. The questionnaires were returned to the research assistants the following day.

### 2.4. Data Analysis

Analyses were conducted using SPSS version 17.0. Frequencies and other descriptive statistics were calculated for all variables. Hierarchical regression analysis was performed to determine the effect of family structure on MVPA of adolescents when gender, age, and SES are controlled.

## 3. Results

### 3.1. Descriptive Analysis

Table 1 presents the descriptive data for the participating students. The ages of the 612 participants ranged from 10 to 16 years (M = 12, SD = 1.2). The gender breakdown was 295 (48.1%) girls and 317 (51.9%) boys. The participants comprised 158 (25.8%) students of Grade 5, 136 (22.2%) students of Grade 6, 178 (29.1%) students of Grade 7, and 140 (22.9%) students of Grade 8 from the seven schools. The Hollingshead Index indicated that 23.1% of families were of high SES, 58.0% were of mid-SES, and 18.9% were of low SES. The participating children spent an average of 22 minutes/day (SD = 14.5) in MVPA based on the Chinese specific cutoff points. A total of 29 (4.7%) adolescents met the recommended 60-minute MVPA per day.

The frequencies of family structure are presented in Table 2. Most adolescents (*n* = 562, 91.9%) reported living with both parents and few (*n* = 44, 7.2%) lived in single-parent households. A total of 514 (84.1%) adolescents reported living with biological parents and a smaller percentage of adolescents (*n* = 92, 15%) lived with step parents. Only six (0.9%) adolescents live with adopted parents or relatives. A total of 498 (81.4%) adolescents reported not residing with either grandparent and 114 (18.6%) participants lived with one or two grandparents. Most adolescents are an only child in their households, and only 42 (6.9%) participants have one sibling.

### 3.2. Multiple Regression Results

Table 3 presents the results of the hierarchical regression analysis by controlling for age, gender, and SES. Model 1 assessed the association among the control factors (i.e., gender, age, and SES) and MVPA duration of the children. Gender was significantly related to the MVPA duration of the adolescents. Boys had longer MVPA duration than girls. However, age was unrelated to the MVPA duration of the adolescents. The middle SES level was negatively associated with the MVPA duration of the adolescents. Middle SES level decreased the MVPA duration of the children compared with those with low SES level. High SES level was insignificantly related to MVPA duration. Gender, age, and SES in this model contributed to 6% of the variance in the MVPA duration of the children (*F*(1, 612) = 10.02, *p* < 0.001).

Family structure is entered in Model 2 and the variance explained in the MVPA duration of the adolescents (*F*(2, 612) = 9.06, *p* < 0.001) increased to 12%. The factors of living with single-parent, with step parents, and with one or two grandparents were significantly related to the MVPA time of the adolescents. Adolescents living with single-parents and with step parents had significantly longer MVPA time compared with those living with both parents and with biological parents, respectively. However, those residing with grandparents were significantly less likely to engage in PA than those not living with either grandparent. No significant differences were found between the MVPA of adolescents living with one sibling and those without siblings.

## 4. Discussion

Public health recommendations for PA have been provided because of the growing awareness of the health benefits of regular PA. The results of the current study indicated that 4.7% of adolescents met the recommendation of 60 minutes of MVPA per day. The results supported previous PA studies that focused on Chinese adolescents [3–5]. This study aims to determine whether an association exists between the family structure and PA participation among adolescents in China. The findings suggest that family structure significantly increased the likelihood of adolescents to engage in PA by controlling for the gender, age, and SES of adolescents. However, this variable only explained 6% of the MVPA duration of the adolescents. This percentage indicates that family structure was not sufficient to explain the MVPA variance because 94% of the variance could not be explained. This result may be because of the following reasons. First, the increased extent of individualization and privatization as well as the relinquishing of traditional norms during the development of modern (or postmodern) society may mean that the younger generation is less dependent on its family background [21]. Second, the low percentage of variance may be explained by early adolescent autonomy (i.e., self-determination, decision-making, and independence) [22]. Autonomy may compel early adolescents to regard less pressure from their families compared to their own personal volition [23, 24].

The present study further examined the relationship between adolescents' MVPA time and different types of families based on different dimensions. The findings of this study are consistent with the study by Sallis et al. [25]; adolescents living with single-parents are more physically active than those with two biological parents. The present study also focused on adolescents living with step parents which has not been examined in previous PA studies and found that these adolescents had significantly longer MVPA time than those who live with biological parents. Several researchers reported that single-parents monitor their children less closely and know less about their whereabouts, whom they are with, and what they are doing compared to parents in intact families [26]. Single-parent families are also more likely to be poor because of the lower earning capacity of a single father or mother and the insufficient benefits provided by the state [27]. Most studies found that step families are less stable, and step parents have fewer resources than two-parent families because they may be providing resources to their prior biological children in other households or because they are less committed to nonbiological children [28–30]. Adolescents from single and step families have less supervision and resources; thus they may have more time to engage in PA, rely on walking or biking for transportation, and do more housework compared to those in two-parent families. Such activities could contribute to their longer MVPA duration [25]. However, insignificant differences were found between adolescents living with both parents and those with neither biological parent and between adolescents living with biological parents and those with adopted parents and relatives. The group of adolescents living with neither biological parent is currently rare and unique in China. Therefore, only six samples in this study live with neither biological parent. The limited sample size could not provide sufficient statistical power to detect such effects and could have led to the insignificant differences in MVPA time between this group of samples and those living with biological parents and with both parents. Further studies with a larger sample size are necessary to examine the PA of adolescents from households with neither biological parent.

The negative association between living with one or two grandparents and MVPA duration of adolescents indicated that adolescents are less physically active when grandparents live with their family compared with adolescents not living with grandparents. This finding may link with the unique Chinese family culture. Many families in China have grandparents who take care of their grandchildren, and thus children spend more time with their grandparents than with their parents [13]. Moreover, grandparents are highly influential in Chinese families, reflecting the Chinese veneration of age and promotion of family values [13, 30]. Thus, the thinking and behavior of grandparents heavily influence the behavior of children. However, some researchers have revealed that grandparents in China tend to overprotect children and that their thoughts agree with Chinese traditions, which highly value academic achievement at the “expense” of PA engagement [31]. These reasons may result in shorter MVPA time of adolescents living with grandparents.

It is surprising that no significant difference was found in MVPA time between adolescents living with one sibling and those without siblings because previous studies reported that siblings influenced PA behavior of children and adolescents [32, 33]. This finding is also different from the study by Bagley et al. who reported that girls with siblings spent more minutes per day in PA compared with girls without siblings [9]. Many families with two or three children have grandparents to take care of the children because parents do not have much time with their children. This phenomenon is consistent with the findings in the present study. More than half of the adolescents living with one or two siblings resided with their grandparents. Having siblings may promote the engagement of PA among children and adolescents; however, the negative influence of grandparents' traditional thoughts on PA may result in the insignificant differences in MVPA time between the sample of adolescents living with one or two siblings and those residing with two parents only.

Except for the family structure, the findings of the present study also indicated that boys had significantly longer MVPA duration than girls. The finding is aligned with a number of previous studies that reported that boys participate in PA at a higher rate than girls [5, 34, 35]. Focusing on SES influence, researchers in Western countries have determined that the higher SES of the family is consistent with the higher PA level of the children [36–38]. Contrary to these findings, the present study revealed that middle SES level was negatively associated with the MVPA duration of the adolescents and the highest SES level was not significantly related to the MVPA duration of the adolescents. Similar results were reported by other Chinese studies [3, 39]. Macintyre and Mutrie explained that, despite higher participation in formal and organized sports of children and adolescents with higher SES, they may participate in less unstructured activities [40]. Shi et al. confirmed these findings under the Chinese context and they determined that Chinese adolescents with low SES did more housework than those with high SES, whereas adolescents with high SES had low percentage of walking to school compared to those with low SES [3]. Less unstructured activities of adolescents with middle SES may lead to shorter MVPA duration when compared with adolescents with low SES level. In addition, Chinese researchers reported that greater economic and educational resources increase parents' investments in both the intellectual and social development of their children in their leisure time [41], which may reduce their time and chances to engage in PA. This factor may provide another explanation for shorter MVPA duration of adolescents with middle SES as compared to those with low SES.

This study is the first study to examine the relationship between special types of family (e.g., living with grandparents and living with step parents) and PA level of adolescents. Certain limitations should be noted although this study has several interesting findings to add to literature. First, this study was a pilot study for a larger, longitudinal investigation. Moreover, the sample size was relatively small, upon attempting to understand behavioral patterns in uncommonly occurring family structures (e.g., living with neither biological parent). Furthermore, the sample was drawn from Shanghai, an international city in China. Thus, whether the pattern of our results is unique to this sample or universal to all Chinese adolescents remains unclear. Future studies would benefit from having a larger sample size obtained nationwide so that it is more heterogeneous and representative of the Chinese society diversity. Second, family structure predicted only 6% of adolescents' PA in the present study. Therefore, other possibly relevant parental variables (i.e., parental support and PA level) or psychological and physical environmental factors are necessary. Third, future studies with larger sample size could consider some more specific factors concerning family structure. For example, the PA level of adolescents living with a single father, a single mother, a father and a step mother, and a mother and a step father may be examined specifically to learn whether differences can be found among the PA levels of these adolescents. Fourth, the cause of parental separation (i.e., divorce, separation, or death of partners) may also be considered. This study did not consider the age and sex of the siblings. Living with older siblings or brother may influence adolescents' PA more than living with siblings and sister. Sibling age and sex are important characteristics to explore in future research. Finally, a qualitative method (i.e., interview) is also necessary to explore how these factors influence the socialization process of adolescents with high levels of PA.

## 5. Conclusion and Implication 

This study was concluded that family structure was significantly associated with the MVPA of adolescents in China. Adolescents living in single-parent households spent more time on MVPA than those living in two-parent homes. Adolescents living with step parents are more physically active compared with those with biological parents. However, adolescents who lived with grandparents were less active than those living with neither grandparent. Therefore, family environment should be considered in the development of PA interventions and policies.

Our findings raise potential implications regarding the promotion of PA among adolescents. The significant relation of family structure to adolescents' PA suggests that this variable plays an important role in adolescents' PA. Therefore, a broader family environment may be an important element to consider in the development of PA interventions and policies. For example, based on our research results, adolescents living with grandparents are a particularly important target group in some intervention programs.

## Figures and Tables

**Table 1:** Descriptive information for study variables (*N* = 612).

Variables	M or *n* (%)	SD	Range
MVPA time	22 min/day	14.5	4–89
Age (in years)	12	1.2	10–16
Gender			
Boys	317 (51.9%)		
Girls	295 (48.1%)		
Grade			
Grade 5	158 (25.8%)		
Grade 6	136 (22.2%)		
Grade 7	178 (29.1%)		
Grade 8	140 (22.9%)		
SES			
Low	116 (18.9%)		
Middle	355 (58.0%)		
High	141 (23.1%)		

**Table 2:** Family structure frequencies (*N* = 612).

Factors	Family structure subcategories (living with)	Frequencies
Number of parents	Single-parent	44 (7.2%)
	Both parents	562 (91.9%)
	No biological parents	6 (0.9%)

Nature of parents	Biological parents	514 (84.1%)
	Step parents	92 (15%)
	Adopted parents and relatives	6 (0.9%)

Number of grandparents	One or two grandparents	114 (18.6%)
	No grandparents	498 (81.4%)

Number of siblings	One sibling	42 (6.9%)
	No siblings	570 (93.1%)

**Table 3:** Summary of hierarchical regression analysis for association between family structure and MVPA time of children (*N* = 612).

Variables	Model 1			Model 2		
	*B*	SE	*β*	*B*	SE	*β*
Age	1.14	0.49	0.08	1.14	0.49	0.09
Girls	−5.62	1.14	−0.19^*∗∗∗*^	−5.48	1.12	−0.19^*∗∗∗*^
SES						
Middle SES	−4.57	1.53	−0.16^*∗∗*^	−3.51	1.51	−0.12^*∗*^
High SES	−2.47	1.75	−0.07	−2.51	1.71	−0.08
Number of parents						
Single-parent				5.75	2.26	0.10^*∗*^
No biological parents				−4.64	6.86	−0.03
Nature of parents						
Step parents				4.88	1.59	0.12^*∗∗*^
Adopted parents or relatives				−2.59	4.03	−0.03
Number of siblings						
One sibling				−4.19	2.29	−0.07
Number of grandparents						
One or two grandparents				−3.30	0.79	−0.17^*∗∗∗*^
*R* ^2^	0.06			0.13		
*R* ^2^ adj	0.06			0.12		

*Note*. ^*∗*^ *p* < 0.05; ^*∗∗*^ *p* < 0.01; ^*∗∗∗*^ *p* < 0.001.

^*∗*^Reference categories are boys, low level of SES, living with both parents, and living with biological parents, living without siblings, and living with neither grandparent.
